# GmMYB181, a Soybean R2R3-MYB Protein, Increases Branch Number in Transgenic *Arabidopsis*

**DOI:** 10.3389/fpls.2018.01027

**Published:** 2018-07-17

**Authors:** Hui Yang, Qian Xue, Zhenzhen Zhang, Jingyi Du, Deyue Yu, Fang Huang

**Affiliations:** National Center for Soybean Improvement, Key Laboratory of Biology and Genetic Improvement of Soybean Ministry of Agriculture, State Key Laboratory of Crop Genetics and Germplasm Enhancement, Nanjing Agricultural University, Nanjing, China

**Keywords:** soybean, MYB transcription factor, GmMYB181, floral organ, branch number

## Abstract

Soybean (*Glycine max*) is an important economic crop that provides abundant oil and high quality protein for human beings. As the process of reproductive growth directly determines the crop seed yield and quality, we initiated studies to identify genes that regulate soybean floral organ development. One R2R3-MYB transcription factor gene, designated as *GmMYB181*, was found to be enriched in flowers based on microarray analysis and was further functionally investigated in transgenic *Arabidopsis*. GmMYB181 protein contains two MYB domains, which localized to the nucleus and displayed transcriptional activation in yeast hybrid system. Real-time quantitative PCR (qRT-PCR) results suggested *GmMYB181* exclusively expressed in flower tissue. In *Arabidopsis*, overexpression of *GmMYB181* altered the morphology of floral organs, fruit size and plant architecture, including outward curly sepals, smaller siliques, increased lateral branches and reduced plant height, indicating that *GmMYB181* is involved in the development of reproductive organs and plays an important role in controlling plant architecture. Further, microarray analysis revealed that overexpressing *GmMYB181* in *Arabidopsis* affected the expression of 3450 genes in mature flowers, including those involved in floral organ, seed/fruit development, and responded to different hormone signals.

## Introduction

Flowering is the most important developmental process in flowering plants, which is determined by environmental and endogenous signals. The floral development undergoes three stages: flowering determination, flower evocation, and floral organs development. The four-whorl floral organs containing sepals, petals, stamens and carpels, are controlled by a group of specific genes, which constitute a genetic “ABCDE” model (Krizek and Fletcher, 2005). The ABCDE model consists of five classes of genes, named as A, B, C, D, and E. A genes alone regulate sepals; A and B genes together control petals; B and C genes determine stamens (Pelaz et al., 2000); C genes alone determine carpels; D genes specify the ovule (Angenent et al., 1995); and E class genes determine the identities of all four types of floral organs (Mandel and Yanofsky, 1998). As is known, gene regulation through TFs has an essential effect on plant growth, organ morphogenesis and stimuli response. To date, several kinds of TFs, such as MADS (Huang et al., 2014), bHLH (Szécsi et al., 2006), bZIP (Sussmilch et al., 2015), NAC (Hendelman et al., 2013; Zhong et al., 2016), Z-C2H2 (Xiao et al., 2009), as well as MYB (Song et al., 2011; Li et al., 2013), have been reported to be involved in plant floral development.

*MYB* genes, encoding one of the largest TF families, are widely present in higher plants. The MYB protein family is defined by a structurally conserved MYB domain and classified into four types depending on the repeat number in the MYB domain: 4R-MYBs (R0R1R2R3-MYB) have four repeats, 3R-MYBs (R1R2R3-MYB) include three repeats, 2R-MYBs (R2R3-MYB) contain two repeats, and 1R-MYB usually contains single repeat or atypical repeats in plant (Dubos et al., 2010). Among the four types, R2R3-type contains the largest number of members and accounts for 70.05% of the *Arabidopsis MYB* gene family (Katiyar et al., 2012), demonstrating that *R2R3*-*MYB* genes constitute the largest subfamily of *MYB* families in plants. Until now, *R2R3*-*MYB* genes in different plant species have been found to be involved in the regulation of floral organ development. In *Arabidopsis, AtMYB21, AtMYB24*, and *AtMYB108*, three JA-inducible genes, were shown to regulate stamen development and male fertility: *AtMYB21* plays a dominant role in the elongation of the stamen filament, and has functional redundancy with *AtMYB24* in the control of anther dehiscence and pollen maturation; *AtMYB108* is required for correct timing of anther dehiscence, and can mediate stamen and pollen maturation when combined with *AtMYB24* (Yang et al., 2007; Mandaokar and Browse, 2009; Song et al., 2011). In addition, another report suggested that *AtMYB21, AtMYB24*, and *AtMYB57*, as the GA-dependent stamen-enriched genes, control stamen filament development via gibberellin and jasmonate interaction (Cheng et al., 2009). The R2R3 MYB TF genes FOUR LIPS (*FLP*) and *AtMYB88* were reported to participate in female reproductive development; loss-of-function of *FLP* and *MYB88* led to reduced female fertility, shorter siliques and reduced seed set (Makkena et al., 2012). *Arabidopsis GAMYB*-like genes *MYB33* and *MYB65*, which can be regulated at the post-transcriptional level by miRNA159, are necessary for anther maturation but not for stamen filament elongation (Millar and Gubler, 2005). In barley, *HvGAMYB* is strongly expressed in anthers and up-regulated by gibberellin (GA); transgenic barley over-expressing *HvGAMYB* gene produced a decrease in anther length and color (Murray et al., 2003). Overexpression of a cotton R2R3 MYB-like gene *GhMYB24* in *Arabidopsis* caused a series of abnormal phenotypes, including deformed flowers, shorter filaments, uncracked anthers, and fewer viable pollen grains (Li et al., 2013). *MdMYB3* is involved in the regulation of anthocyanin biosynthesis and floral pistil development in apple (*Malus domestica Borkh*.) (Vimolmangkang et al., 2013). *VvMYB5b* from grapevine affects reproductive development when transformed into tomato, exhibiting abnormal floral organs, smaller fruit and darker brown seeds (Mahjoub et al., 2009).

As is known, shoot (vegetative) and inflorescence branching are initiated during post-embryonic development and formed by the secondary meristems in seed plants, which will determine the architecture and reproductive growth of a plant (Schmitz and Theres, 2005). The MYB TFs have been studied as the key regulators of early steps during shoot branching process, which is controlled by the regulatory mechanism involving auxin (Schmitz and Theres, 2005). *Blind* gene, encoding a R2R3-MYB TF, has been reported to control the lateral meristem initiation in tomato: RNA interference of tomato *Bl* resulted in decreases in lateral branch number and the flower number per inflorescence (Schmitz et al., 2002). Similarly, three *Arabidopsis RAX* (1-3) genes, the homologs of *Blind*, are partially redundant in function and regulate early steps of axillary meristem initiation (Müller et al., 2006).

Soybean (*Glycine max*) is an important source of edible oil and vegetable protein in the world and plays a significant part in agriculture and economy. There were in total 252 *MYB* genes identified in soybean, comprising 244 *R2R3*-*MYB* (2R-MYBs) genes, six *R1R2R3*-*MYB* (3R-MYBs) genes, and two *R0R1R2R3*-*MYB* (4R-MYBs) genes (Du et al., 2012). Until now, fewer studies have been reported about the roles of *MYB* members in soybean, especially about the function of flower development. Takahashi et al. (2013) determined that *GmMYB-G20-1* might correspond to the W2 gene that produced purple-blue color and high vacuolar pH of flower petals in soybean (Takahashi et al., 2013). In this study, we identified a soybean *R2R3*-*MYB* type gene, *GmMYB181*, and characterized its expression pattern, protein localization, and transcriptional activation. Overexpressing *GmMYB181* in *Arabidopsis* increased the lateral branches, determined floral organ formation and fruit size. We then attempted to explain the mechanism by which *GmMYB181* affects the reproductive development and plant architecture in transgenic *Arabidopsis* plants using microarray analysis. This work presents a basic function and mechanism understanding of soybean *GmMYB181*, which will lay the foundation for further deep research of *GmMYB181* in soybean.

## Materials and Methods

### Plant Materials and Growth Conditions

The soybean cultivar (Monkey hair) was sown in the field of Jiangpu experimental station, Nanjing Agricultural University. Different tissues containing roots, stems, leaves, flowers, seeds, and pod shells were collected for qRT-PCR analysis of *GmMYB181*. These tissues were, respectively, sampled at different developmental stages: roots, stems, and leaves were from seedling stage; mature flowers were from flowering stage; seeds and pod shells were obtained at 7, 15, 25, 40 DAF.

*Arabidopsis thaliana* (ecotype Columbia-0) was used as WT. Seeds were sown on solid MS medium after incubation (48–72 h at 4°C) and sterilization, and then were put in a growth room under the condition of 16/8 h light/dark, 23/22°C, with 70% relative humidity.

### Cloning of *GmMYB181* Gene

The full-length cDNA of *GmMYB181* (GeneBank Accession No. DQ822906) was PCR-amplified from the flower cDNA of soybean cultivar monkey hair (primers shown in Supplementary Table S1). The PCR products were gel-purified (Axygen, United States), and cloned into the pMD19-T vector (TaKaRa, Dalian, China) for sequencing (Invitrogen, Shanghai, China).

### Gene Expression Analysis

Digital tissue expression pattern of *GmMYB181* in soybean was examined using SoyBase database^1^ and soybean eFP Browser^2^.

Total RNA was extracted from soybean and *Arabidopsis* with Plant RNA Extract Kit (TianGen, Beijing, China) and cDNA was reverse transcribed using PrimeScript^TM^ 1st Strand cDNA Synthesis Kit (TaKaRa, Dalian, China). qRT-PCR was conducted with SYBR^®^ Green Real-time PCR Master Mix (Toyobo, Japan) on a iQ5 real-time PCR instrument (Bio-Rad, United States). SqPCR was carried out with 2 × Taq PCR MasterMix (TaKaRa, Dalian, China). All primer pairs used are listed in Supplementary Tables S1, S2. Soybean internal genes including *actin* (*Glyma.04G215900*) and *tubulin* gene (GenBank Accession No. AY907703) were used for SqPCR and qRT-PCR, respectively. *Arabidopsis tubulin* gene (*AT5G62690*) was used as internal control. The relative expression levels of *GmMYB181* were calculated utilizing the 2^-ΔΔCt^ method (Livak and Schmittgen, 2001).

### Sequence and Phylogenetic Analysis

Gene structure was analyzed using GSDS^3^. The characteristics of GmMYB181 protein were analyzed by BioXM program (ver 2.6), and the conserved domains were identified with SMART^4^. The protein sequences of *MYB* genes (accession numbers are listed in Supplementary Table S3) from different plants were obtained from NCBI databases^5^. Sequence alignment was performed with ClustalX 2.0 program and viewed with GeneDOC. A NJ phylogenetic tree was constructed based on protein sequences with MEGA 6.0 software using bootstrap method with 1,000 replications.

### Subcellular Localization Analysis

The TargetP software^6^ was employed to predict the subcellular localization of GmMYB181. The CDS of *GmMYB181* without the termination codon was cloned into the pMDC83-GFP vector to generate the construct 35S:GmMYB181:GFP. This gene construct and control (empty vector 35S:GFP) were, respectively, transferred to onion epidermal cells through particle bombardment method. The images were photographed by confocal laser-scanning microscopy (Leica TCS SP2, Mannheim, Germany).

### Transcriptional Activity Assay

The transactivation activity analysis of GmMYB181 was conducted by utilizing yeast two-hybrid system. For BD-GmMYB181 construct, the coding region of *GmMYB181* was cloned into pDEST32 vector by LR reaction using Gateway^®^ Technology with Clonase^TM^ II (Invitrogen). BD-GmMYB181 and AD (pDEST-22), positive (pEXP32-Krev and pExP22-Ra1GDS-wt) and negative control (pEXP32-Krev1and pEXP22-Ra1GDS-m2) vectors were, respectively, introduced into yeast strain MaV203, which were then grown on SD/-Leu/-Trp/-His + 40 mM 3-amino-triazole (3-AT) plates for at least 2 days at 30°C.

### *Arabidopsis* Transformation

The CDS of *GmMYB181* was amplified with specific primers (Supplementary Table S1) and fused into pMDC83 vector by Gateway^TM^ Technology (Invitrogen, Shanghai). Then the recombinant plasmids were transformed into *Arabidopsis* using the floral dip method (Mara et al., 2010). *GmMYB181* transgenic *Arabidopsis* plants were first screened on MS medium containing 50 μg ml^-1^ HygB, and then resistant plants were examined on genomic and transcriptional levels. Leaf genomic DNA was extracted using CTAB method for PCR analysis; flower total RNA were extracted for SqPCR and qRT-PCR analyses.

### *Arabidopsis* Phenotype Investigation

Wild type and three *GmMYB181* transgenic lines were used to identify the phenotype differences. The branch number was calculated at 28, 35, 43, 51 DAT with 15 seedlings (each line). Silique length and seed number were measured with 10 seedlings (each line), and 10 siliques (each seedling). Paired-samples *t*-test (two-tail) was used for statistical analysis between WT and transgenic *Arabidopsis* plant. ^∗^0.01 < *P* < 0.05; ^∗∗^*P* < 0.01.

### Microarray Analysis of Gene Expression

Mature flower tissues from wild and *GmMYB181* transgenic *Arabidopsis* plants were collected (three biological replicates) at full-bloom stage for RNA isolation. Further, total mRNAs were hybridized on Agilent *Arabidopsis* (V4) Expression 4 × 44K Microarray Chip by CapitalBio Technology. Then, the arrays were scanned by using Agilent G2565CA Microarray scanner (Agilent Technologies) and images obtained were saved as.tiff pictures. Feature Extraction software was used to convert the image signals into digital signals. The data normalization was achieved by the 75th percentile shift method with GeneSpring GX software. Significantly DEGs between WT and *GmMYB181* transgenic plants were obtained based on the following criteria: FC(abs) = |log_2_FC|≥ 2 (FC represented fold change of expression between wild and transgenic *Arabidopsis* plants) and *P*-value < 0.05.

Gene ontology was analyzed with agriGO database^7^ and KEGG pathway was performed by KOBAS 2.0 program^8^. The significantly enriched GO terms and KEGG pathways were selected according to *P*-value after FDR correction < 0.05.

### Bioinformatics Prediction of Regulatory Factors of *GmMYB181*

Plant CARE database^9^ was used to predict the *cis-*acting elements in the promoter region (1500 bp sequence upstream of ATG) of *GmMYB181*. Plant TF Database^10^ was used to evaluate the TFs regulating *GmMYB181*. psRNATarget program^11^ was utilized to identify the microRNA regulating *GmMYB181*.

## Results

### Isolation and Sequence Analysis of *GmMYB181*

**FIGURE 1:**
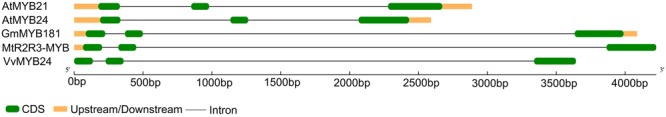
Gene structure analysis of plant *MYB* genes. Plant *MYB* genes contain soybean *GmMYB181, Arabidopsis AtMYB21* and *AtMYB24, Medicago truncatula MtR2R3-MYB* and Grape *VvMYB24*. The green boxes and black lines represent exons and introns, respectively. The orange boxes indicate both upstream and downstream regions.

**FIGURE 2:**
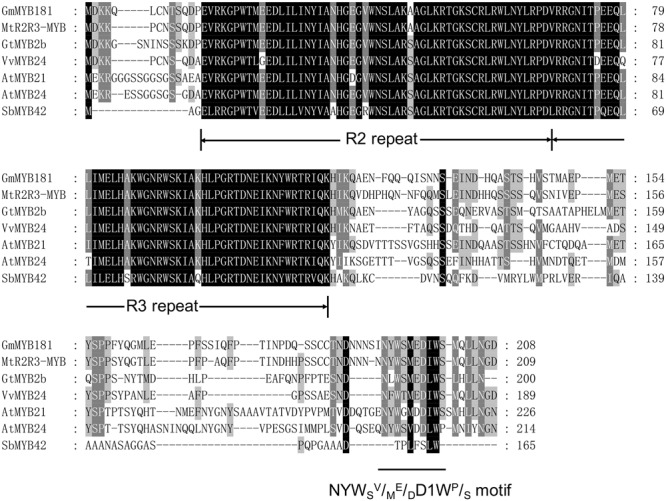
Amino acid sequence alignments. Full protein sequences of soybean GmMYB181 and other R2R3 MYB homologs (accession numbers are listed in Supplementary Table S3) from *Medicago truncatula, Gentiana triflora* Pall, Grape, *Arabidopsis thaliana*, and *Sorghum bicolor*. The conserved R2 and R3 domains and the NYWSV/ME/DDIWP/S motif are underlined.

**FIGURE 3:**
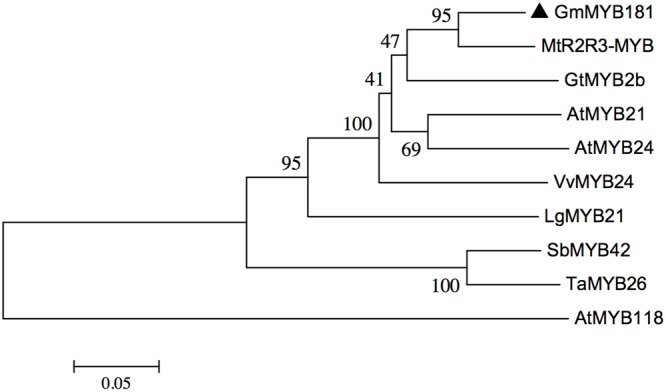
Phylogenetic analysis of *GmMYB181* and other plant *R2R3 MYB* genes. A NJ phylogenetic tree was constructed using full protein sequences (accession numbers are listed in Supplementary Table S3) with MEGA 6.0 software. Numbers below branches indicate bootstrap value for 1000 replicates.

Through microarray analysis, we identified a number of flower-enriched genes. One gene (Gma.18002.1.S1_at) encoding MYB TF designated as *GmMYB181* in NCBI GenBank (Accession No. DQ822906) was selected for further functional study. The cDNA sequence of *GmMYB181* was amplified by RT-PCR from soybean flower (Supplementary Figure S1), which is 750 bp in length and contains an ORF of 627 bp (Supplementary Figure S2). Gene structure analysis indicated that *GmMYB181* contains three exons and two introns (**Figure 1**). By comparing the gene structures of *GmMYB181* and other plant R2R3-MYB type genes, we detected that *GmMYB181* and other plant R2R3-MYB type genes have the same number of exons and introns but divergent in gene sizes, which is mainly due to the variable intron sizes (**Figure 1**). GmMYB181 protein is composed of 208 amino acids with molecular mass of 51.10 kDa and PI of 4.99. Further, GmMYB181 protein was predicted to have two conserved MYB domains (Supplementary Figure S2), which is the typical feature of R2R3-MYB subfamily. The two MYB domains, R2 and R3, comprise of 51 (12–64) and 49 (65–119) amino acids, respectively (Supplementary Figure S2). Alignments of the GmMYB181 protein sequence with other plant MYB proteins showed that GmMYB181 is homologous to *Medicago truncatula* R2R3-MYB (identity of 84%), Grape VvMYB24 (identity of 72%), *Gentiana triflora* Pall GtMYB2b (identity of 71%), *Arabidopsis* AtMYB21 (identity of 63%) and AtMYB24 (identity of 61%) (**Figure 2**). All the plant MYB proteins examined contained conserved N-terminal R2R3 domains, which are presumably required for DNA binding (**Figure 2**). Moreover, they contained NYWs^V^/_M_^E^/_D_DlW^P^/_S_ motif at the C-terminus while showed high divergence (**Figure 2**), which is consistent with a previous analysis in *Arabidopsis* that the subgroups of R2R3 MYB family showed limited sequence conservation within their C-terminal regions (Kranz et al., 1998). To evaluate the relationships of GmMYB181 with other plant MYB proteins, we also built a NJ phylogenetic tree using the amino acid sequences (**Figure 3**). The tree suggested GmMYB181 grouped together with MtR2R3-MYB, VvMYB24, GtMYB2b, AtMYB21, AtMYB24, and LgMYB21 from *M. truncatula*, Grape, *G. triflora* Pall, *Arabidopsis* and *Linum grandiflorum*. Among these orthologs, *VvMYB24* is highly expressed in inflorescences at pollination stage (Matus et al., 2008); *AtMYB21* and *AtMYB24* have a role in the stamen development process (Song et al., 2011); overexpression of *LgMYB21* in *Arabidopsis* led to decreased pistil length and shortened stamens (Ushijima et al., 2012). Since the majority of genes with similar functions tend to cluster on a phylogenetic tree, the role of *GmMYB181* can be inferred from their homologs.

### GmMYB181 Is a Nuclear Protein

**FIGURE 4:**
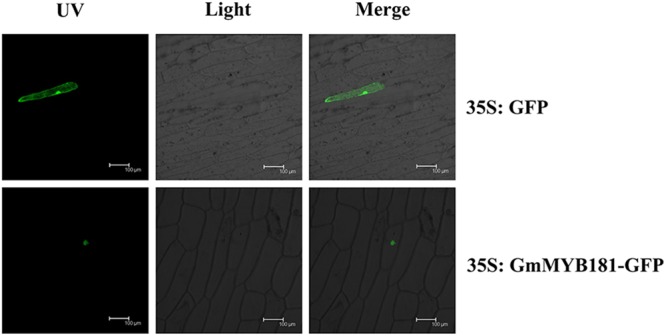
Subcellular localization of GmMYB181 protein in onion epidermal cells. UV, GFP fluorescence; Light, bright field; Merge, combination. Scale bars: 100 μm.

Based on the TargetP software, GmMYB181 was predicted to be localized in the nucleus. In order to examine the localization of GmMYB181 *in vivo*, we cloned the CDS of *GmMYB181* into pMDC83-GFP vector and obtained the construct 35S:GmMYB181-GFP. Confocal images indicated that GmMYB181-GFP fusion protein was exclusively distributed in the nucleus of onion epidermal cells (**Figure 4**).

### GmMYB181 Exhibits Transactivation Activity

*GmMYB181* CDS was cloned into the GAL4 DNA-BD vector pDEST32. The fusion plasmid pDEST32-GmMYB181 and pDEST-22 empty vector [containing GAL4 AD] were transformed into yeast strain MaV203, and then screened by selective medium (Supplementary Figure S3). As shown in Supplementary Figure S3B, the growth of yeast cells containing the negative control vector was significantly inhibited on the selective medium of SD/-Leu/-Trp/-His + 40 mM 3-amino-triazole (3-AT) compared with that on YPAD medium (Supplementary Figure S3A). However, the yeast cells with *GmMYB181* fusion plasmid or the positive control vector grew well on the same selective medium (Supplementary Figure S3B), indicating that GmMYB181 possesses transactivation ability in yeast.

### *GmMYB181* Had Specific Expression in Flower Organ

**FIGURE 5:**
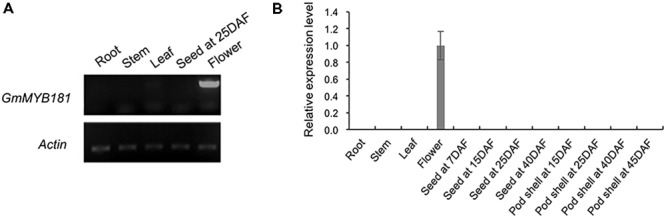
Tissue expression analysis of *GmMYB181*. **(A)** SqRT-PCR and **(B)** qRT-PCR analysis of *GmMYB181* in vegetative organs, flower, seed, and pod at different developmental stages. Gene expression in flower organ was used as control (expression value = 1). DAF, day after flowering.

According to the SoyBase RNA-seq data^12^ (Supplementary Figure S4A) and the soybean eFP Browser^13^ (Supplementary Figure S4B), *GmMYB181* is particularly expressed in flowers, indicating that it may regulate flower development-related trait. We then examined its transcripts in soybean various tissues/organs at different developmental stages using SqRT-PCR (**Figure 5A**) and qRT-PCR (**Figure 5B**). The results showed that the transcripts of *GmMYB181* were only present in flower organs, not in other tissues, suggesting that *GmMYB181* is a flower-specific gene (**Figure 5**).

### Overexpression of *GmMYB181* in *Arabidopsis* Altered the Flower Morphology, Silique Length, and Branch Number

The coding region of *GmMYB181* was inserted into pMDC83 vector to get the construct 35S:GmMYB181, and then the recombinant construct was transferred into the *Arabidopsis* using *Agrobacterium*-mediated transformation method. The 35S:GmMYB181 transgenic plants were screened on MS medium containing 50 μg ml^-1^ HygB (Supplementary Figure S5) and were examined by PCR (Supplementary Figure S6A) and SqPCR (Supplementary Figure S6B) and qRT-PCR (Supplementary Figure S6C) at genomic and transcriptional levels, respectively.

**FIGURE 6:**
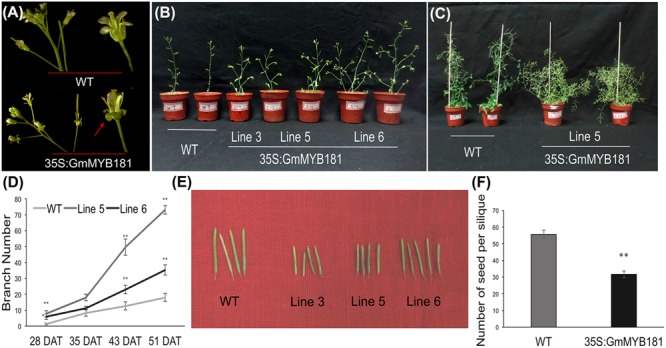
Phenotypic characterization of 35S:GmMYB181 *Arabidopsis* plants. **(A)** Floral organ morphology; the red arrow indicates outward curly sepals in transgenic plants. **(B)** Branching phenotype of 35S:GmMYB181 transgenic and WT plants at 28 DAT (days after transplanting). **(C)** Plant architecture of *GmMYB181* transgenic and WT plants close to maturity. **(D)** Branch number statistics of two genotypes of plants during whole growth period. **(E)** Comparison of silique length between transgenic and WT plants. **(F)** Comparison of seed number per silique between transgenic and WT plants. Paired-samples *t*-test (two-tail) was used for statistical analysis. ^∗^0.01 < *P* < 0.05; ^∗∗^*P* < 0.01.

Compared with WT plants, all transgenic lines from T_2_ and T_3_ generations showed a series of altered phenotypes: (1) we first observed the floral organs and found that there were no any phenotype changes in the flower buds between WT and transgenic *Arabidopsis* plants; however, when the petals fully expanded, *GmMYB181* transgenic plants obviously exhibited outward curly sepals compared with WT (**Figure 6A**). (2) In their subsequent growth, overexpressing *GmMYB181* resulted in dwarf plants and increased lateral branches (**Figures 6B,C**). Therefore, we measured the total branch number of both WT and transgenic plants during their whole growth period (**Figure 6D**), the results indicated that all plants were gradually producing shoot branches at seedling stage, with several more branches in *GmMYB181* overexpression plants compared with WT. There was comparable branch number between transgenic and WT plants near flowering [nearly 35 DAT]; then inflorescence branching began to increase during their reproductive development, and *GmMYB181* transgenic plants still obtained many more branches compared with WT plants. (3) At maturity, we detected that *GmMYB181*-overexpressing plants’ silique lengths were significantly shorter than WT (**Figure 6E** and **Table 1**). To analyze whether the short siliques would affect the seed characteristics, we used T_3_ transgenic and WT plants to measure the seed number (each genotype contain three lines and each line contain 10 siliques). The results showed that the number of seeds per silique of transgenic plants was significantly reduced compared with the WT (**Figure 6F**).

**Table 1:** Silique length of T_2_ *GmMYB181* transgenic and WT *Arabidopsis* plants.

Genotype	35S:GmMYB181			WT
	Line 3	Line 5	Line 6	
Single silique length (cm)	0.87 ± 0.091^∗∗^	0.94 ± 0.095^∗∗^	1.02 ± 0.097^∗^	1.22 ± 0.090

*Lines 3, 5, 6 represent different transgenic lines; WT represents wild type *Arabidopsis thaliana*. 100 siliques were measured in each line.*

### Microarray Analysis of Wild Type *Arabidopsis* and *GmMYB181* Transgenic Plants

**FIGURE 7:**
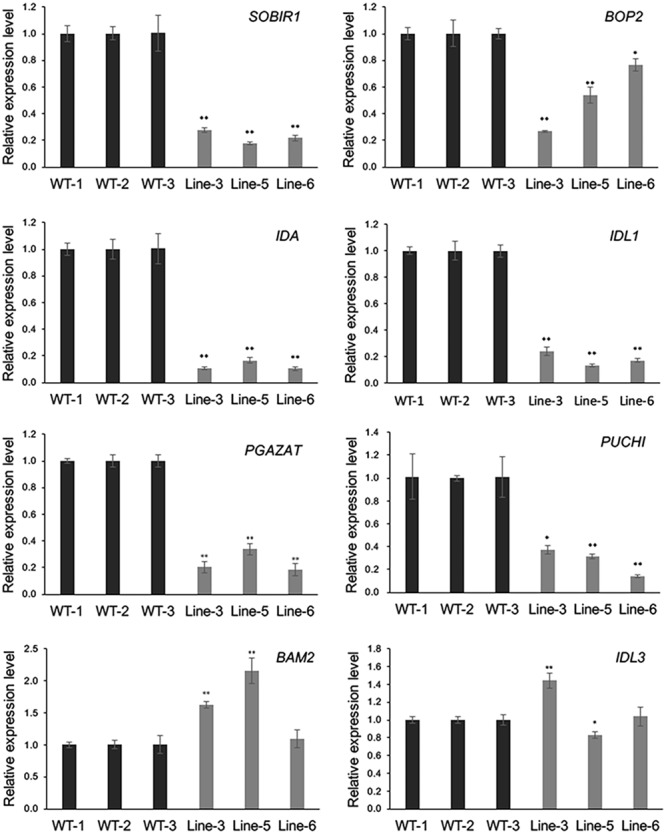
Microarray data confirmation. Eight DEGs were selected for confirmation in flower tissue by using qRT-PCR. Gene expression in WT was used as control (expression value = 1). Paired-samples *t*-test (one-tail) was used. ^∗^0.01 < *P* < 0.05; ^∗∗^*P* < 0.01.

As *GmMYB181* overexpression resulted in abnormal phenotype changes in *Arabidopsis* flower organs, microarray was used to evaluate the mRNA levels in the flowers of WT and *GmMYB181*-overexpressing *Arabidopsis* plants. The Pearson’s correlation coefficient (R) between three biological replicates from microarray was found to be ≥ 0.97 (Supplementary Figure S7). In total, there were 3450 significantly DEGs identified between WT and transgenic *Arabidopsis* plants based on the criteria of *P*-value < 0.05 and |log_2_FC|≥ 2, of which, 1222 were up-regulated and 2228 were down-regulated (Supplementary Table S4). Further, eight genes were selected to validate the reliability of the microarray results through qRT-PCR (**Figure 7**). These eight genes encode some proteins and TFs that are involved in floral organ development. From the results, the relative expression levels of most examined genes generally agreed with the microarray data except for one gene *IDL3* (A_84_P768403), which had different expression changes in three transgenic lines compared with WT (**Figure 7**). *SOBIR1* (A_84_P18180), *BOP2* (A_84_P291394), *IDA* (A_84_P610349), *IDL1* (A_84_P612395), *PGAZAT* (A_84_P861846) and *PUCHI* (A_84_P20594), were all down-regulated in *GmMYB181*-overexpressing plants compared with WT. *BAM2* (A_84_P12759) was up-regulated in *GmMYB181*-overexpressing plants.

### *GmMYB181* Alters the Expression of Many Genes Involved in Flower, Seed, and Hormone Response in *Arabidopsis thaliana*

**FIGURE 8:**
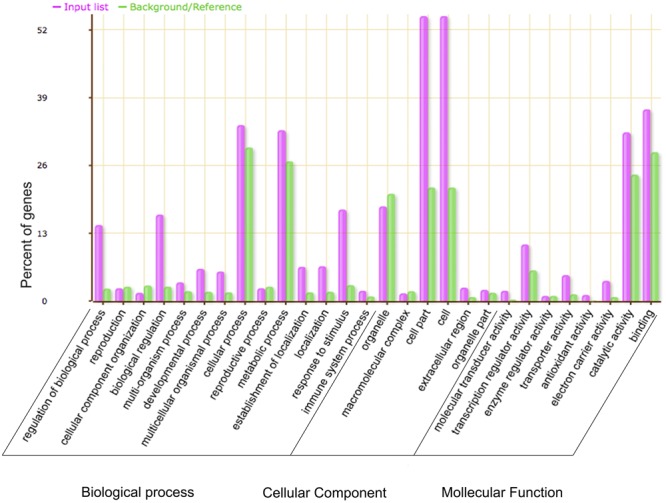
Gene Ontology analysis of the DEGs between 35S:GmMYB181 transgenic and WT *Arabidopsis* plants. The X-axis is the definition of GO terms, and Y-axis is the percentage of genes mapped by the GO term. The pink represents “input list” and refers to DEGs; the green represents “background or reference,” which means background genes. The percentage for the input list is calculated by the number of DEGs mapped to the GO term divided by the number of all DEGs in the input list. The same calculation was applied to the reference list to generate its percentage.

Gene ontology analysis was used to investigate the potential functions of the DEGs between WT and the *GmMYB181*-overexpressing *Arabidopsis* plants. A total of 2199 DEGs were annotated and significantly enriched in 337 GO terms (corrected *P*-value < 0.05) (Supplementary Table S5). As shown in **Figure 8**, the majority of the DEGs were associated with “biological regulation,” “metabolic process,” “response to stimulus,” “cell part” and “catalytic activity.” 35 genes related to flower development were significantly induced in transgenic plants compared to the WT: many genes related to pollen or anther growth including *ACOS5* (A_84_P24111), *AGL104* (A_84_P17192), *BAM2* (A_84_P12759), *CYP703A2* (A_84_P12424), *MS1* (A_84_P565469), *MS2* (A_84_P13673), *MSP2* (A_84_P515906), *QRT3* (A_84_P10038), and *TDF1* (A_84_P16498) were up-regulated in transgenic plants; several genes involving floral organs abscission, floral meristem identity and flowering time including *SOBRI* (A_84_P18180), *BOP2* (A_84_P291394), *IDA* (A_84_P610349), *IDL1* (A_84_P612395), *PGAZAT* (A_84_P861846), *PUCHI* (A_84_P20594), *CO* (A_84_P265780), *RAV1* (A_84_P13235), *COL1* (A_84_P16960), and *COL2* (A_84_P20292) were down-regulated in transgenic plants. These results suggested that overexpression of soybean *GmMYB181* affected the expression of some flower-related genes in *Arabidopsis*. Moreover, some DEGs involved in seed/fruit development were induced in *GmMYB181* overexpression plants, like *MYB5* (A_84_P19329) and *MYB61* (A_84_P89429), *NAP* (A_84_P14306), *SHB1* (A_84_P12896), *TTG2* (A_84_P22046), and *MINI3*/W*RKY10* (A_84_P22807).

Except for these genes mentioned, many of the 2199 affected genes are involved in vegetative growth, metabolism, stimulation, signaling, cellular transporters, cell wall synthesis and the cytoskeleton, which might be important for the phenotypes of *Arabidopsis* transgenic plants. For example, IAA31, the *Arabidopsis* Aux/IAA protein, regulates auxin-related phenotypes including plant height, gravitropic growth orientation, cotyledons, and root development (Sato and Yamamoto, 2008). AtCCD7 and AtCCD8, the cleavage deoxygenase of carotenoids, are expected to catalyze the synthesis of a carotenoid-derived signal molecule, which is a necessary condition for lateral branch regulation (Schwartz et al., 2004). *PIN5*, encoding a functional auxin transporter, mediates auxin homeostasis at the ER, and cell-to-cell auxin transport at the plasma membrane (Mravec et al., 2009). MIF1, a putative zinc finger protein from *Arabidopsis*, is involved in regulating plant development through multiple hormones (Hu and Ma, 2006).

**FIGURE 9:**
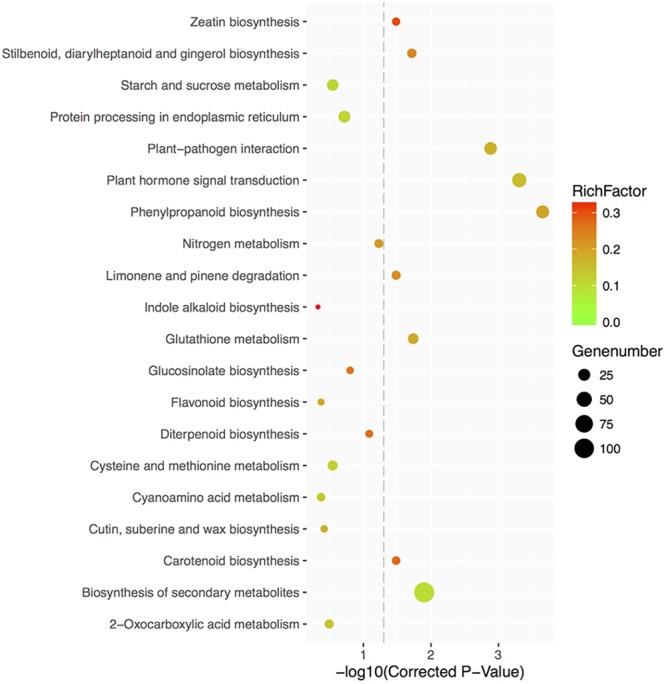
Top 20 KEGG pathways enrichment scatter diagram of 35S:GmMYB181 overexpressed *Arabidopsis* plants. The X-axis represents the enrichment degree, which is determined by the correct *P*-values; the Y-axis represents the pathway terms; the color of the spots indicates the enrichment factor, which represents the ratio of number of DEGs compared with genome background in a pathway, and the spots size represents the number of significant DEGs. The picture was drawn by local program.

Furthermore, 325 DEGs were categorized into 99 relevant KEGG pathways, of which nine were significantly enriched (corrected *P*-value < 0.05) (**Figure 9** and Supplementary Table S6). Eight of the 9 KEGG pathways were found to be associated with metabolic processes. Among the 8 pathways, 31 DEGs are involved in phenylpropanoid biosynthesis, 42 in plant hormone signal transduction, 107 in biosynthesis of secondary metabolites, 17 in glutathione metabolism, 11 in stilbenoid, diarylheptanoid and gingerol biosynthesis, 8 in carotenoid biosynthesis, 10 in limonene and pinene degradation, and 7 in zeatin biosynthesis. Floral organ regulators, like *ACOS5*, were enriched in phenylpropanoid biosynthesis, biosynthesis of secondary metabolites, ubiquinone and other terpenoid-quinone biosynthesis and metabolic pathways; *CO*, controlling flowering time, was categorized in plant circadian rhythm pathway. Several DEGs involved in branching and hormone responses were associated with plant hormone signal transduction, like *AtCCD8* (carotenoid biosynthesis pathway), *IAA31* and *ABF3*.

Altogether, ectopic expression of *GmMYB181* affects the expression of many genes that are classified as developmental process-related proteins, biosynthesis- and metabolism- related proteins, transporters, cellular process-related proteins and catalytic activity proteins. Therefore, these DEGs might be the reason causing the changes in the phenotypes in reproductive organs and plant architecture of *GmMYB181* transgenic *Arabidopsis* plants.

### Characterization of Putative Regulatory Factors of *GmMYB181*

As is known, most TFs regulate the target genes, moreover, their own activities are regulated by other protein factors at various levels. To investigate the TFs regulating *GmMYB181* at transcriptional level, we analyzed the *cis-*elements in the promoter sequence of *GmMYB181* and predicted the TFs that might regulate *GmMYB181*. A total of 29 *cis-*acting elements (Supplementary Table S7) were predicted in the 1500 bp upstream of *GmMYB181* via PlantCARE database, including AAAC-motif (light responsive element), CCAAT-box (MYBHv1 binding site), CGTCA-motif (MeJA-responsive element), TCA-element (salicylic acid responsive element), LTR (low-temperature responsive element), RY-element (seed-specific regulatory element), Skn-1_motif (endosperm expression), TC-rich repeats (defense and stress responsive element) and circadian (circadian controlling element). Then we predicted 24 putative regulatory TFs (Supplementary Table S8) by using Plant TF Database, which belong to the gene families involving MYB, C2H2, C3H, B3, TALE, bzip, EIL, AP2, BBR-BPCT, and Dof. Among them, *GmBBM1* (*Glyma.09G248200*) from AP2 family, is reported to regulate somatic embryogenesis and embryo development in transgenic *Arabidopsis* (Ouakfaoui et al., 2010).

Post-transcriptional regulation also has important influences on gene expression in eukaryotes, for example microRNA regulation. MicroRNA (miRNA) is a class of endogenous non-coding small RNA (sRNA) with 19–24 nucleotide (nt), which usually repress the target gene expression through splicing mRNA or inhibiting mRNA translation (Rogers and Chen, 2013). By using psRNATarget program, three miRNAs containing gma-miR2108a, gma-miR4359a and gma-miR5035-5p, were predicted to target *GmMYB181* (Supplementary Table S9). However, these three miRNAs haven’t been studied until now, thus their functions toward *GmMYB181* require more experimental verification.

## Discussion

MYB TF is a large family that regulates various plant growth and development processes. Until now, there are about 17 members of this family in soybean have been reported. For instance, one particular seed coat-expressed R2R3 MYB gene (*Glyma09g36990*) is identified to affect the brown seed coat/hilum phenotype in soybean based on fine mapping (Gillman et al., 2011). GmMYBJ1, a typical R2R3-MYB protein, responds to abiotic stresses, including drought, cold, salt and abscisic acid (ABA), and confers drought and cold tolerance in *Arabidopsis* (Su et al., 2014). GmMYB73, homolog of CPC-like MYB protein, elevated the lipid contents in both seeds and leaves of transgenic *Arabidopsis* and *Lotus*, and in transgenic hairy roots of soybean plants (Liu et al., 2014). However, relatively few *MYB* genes of soybean have been studied in development especially reproductive development. A previous study in our lab that investigation of genome wide expression profiles by Affymetrix Gene Chip in different tissues (Huang et al., 2009) revealed three soybean *MYB* genes that are mainly expressed in flower. In this study, one of these three genes, named as *GmMYB181*, encoding a R2R3-type MYB transcriptional factor, was selected for functional characterization.

Many reported MYB proteins are presumed to be transcriptional activators. For instance, *BjMYB1* from *Brassica juncea* potentially participates in host defense by activating the expression of *BjCHI1* through the binding of Wbl-4 element in BjC-P promoter (Gao et al., 2016). *MtMYB3* was identified to directly bind the MYB *cis-*elements in the promoter of *MtCBF4*, which could enhance drought and salt tolerance in *M. truncatula*, leading to the inhibition of *MtCBF4* expression (Zhang et al., 2016). Our result suggested that soybean GmMYB181 has transcriptional activation activity in the yeast system, which needs to be further examined in the plant system.

Based on blastp analysis with the MYB proteins from other plants, we obtained several known *MYB* genes including *VvMYB24* from Grape (Matus et al., 2008), *LgMYB21* from *L. grandiflorum* (Ushijima et al., 2012), *AtMYB21* and *AtMYB24* (Song et al., 2011) from *Arabidopsis* that were closely clustered with *GmMYB181*, indicating that *GmMYB181* is very likely to regulate soybean reproductive development. Both digital and experimental tissue expression results showed that *GmMYB181* was only expressed in the flower organ, but not in other tissues, even in other reproductive organs such as seeds and pod shells. Thus, we predicted that *GmMYB181* might be involved in floral organ development.

Further, *GmMYB181* was functional analyzed by transforming into *Arabidopsis*. A total of nine transgenic lines were obtained, but they exhibited different expression levels via qRT-PCR examination: lines 3, 5, and 6 had relatively high expression levels while the remaining lines displayed much lower expression, such as lines 1, 4, and 7. These phenomena might be related to genomic position effect and gene copy number. As the insertion and integration sites of exogenous genes on the genome are random, thus the insertion site property and its collateral sequences may have influences on the activity of exogenous genes, which is called position effect (Chandler and Vaucheret, 2001; Kim et al., 2007). In general, a transgene integrated into the heterochromatin region tends to be silenced, while integration into the transcription-rich euchromatin region tends to be expressed. In addition, many studies demonstrated that the inactivation of a site is positively correlated with the copy number of inserted exogenous genes (Tang et al., 2003). Compared with WT, 35S:GmMYB181 transgenic lines exhibited outward curly sepals when close to flowering, accompanied by other morphological variations in comparison with wild type plants including shorter siliques, reduced seed number, dwarf plants and increased lateral branches. Sepals can protect the developing reproductive organs before the flowers bloom, also lateral branching and silique length will ultimately affect yield traits. Therefore, these results suggested that *GmMYB181* may play important roles in the entire developmental processes of *Arabidopsis* plants. Though, some similar phenotypes are found in other researches: *Arabidopsis FLP* and *AtMYB88* were reported to regulate female fertility, silique length and seed set (Makkena et al., 2012); grapevine *VvMYB5b* affects floral organ, fruit size and seed color when transformed into tomato (Mahjoub et al., 2009), there is no one *MYB* gene reported like *GmMYB181* that regulates developments both in plant reproduction and architecture. The functional analysis of *GmMYB181* in transgenic *Arabidopsis* provided us a reference for the future in-deep study for floral organ development via soybean transformation system.

A total of 3450 significantly DEGs were identified between WT and transgenic *Arabidopsis* plants based on our microarray assay. Among them, some DEGs might be directly or indirectly regulated by *GmMYB181*. However, there possibly existed some other DEGs that were caused by the position effect of *GmMYB181*. Since the genome insertion sites of exogenous genes could change the bases composition in their nearby regions, some of plant endogenous genes would be methylated; in addition, the integration of exogenous genes might cause some degree of gene rearrangement, which will influence the expression and inheritance of plant endogenous genes (Stam et al., 1997; Kumar and Fladung, 2001). In this study, since the similar phenotypes were observed in three independent *GmMYB181* transgenic lines, the alterations of the plant architecture in transgenic *Arabidopsis* were indeed conferred by GmMYB181.

Many processes are required for the normal vegetative and reproductive growth, such as biological regulation, metabolic process, signaling, catalytic activity, cellular transport, cell wall synthesis and the cytoskeleton, among others. Overexpression of *GmMYB81* significantly altered the expression of many genes that are involved in vegetative growth (branch and plant height), floral organ, and seed/fruit development, and response to several hormone stimulus. We found that *ACOS5* and *CYP703A2* were both significantly up-regulated in the *GmMYB181 Arabidopsis* transgenic plants. The *ACOS5* gene encodes an Acyl-CoA synthetase protein, and the *acos5* mutants exhibit a severe defect in pollen tube growth, thus producing no seeds by self-fertilization (Souza et al., 2009). CYP703A2 is a cytochrome P450 family protein related to saturated fatty acids catalysis in growing anthers; knockout of *Arabidopsis CYP703A2* resulted in impaired pollen and a partial male-sterile phenotype (Morant et al., 2007). In addition, several genes involved in floral organs abscission (e.g., *BOP2* and *IDA*) were significantly down-regulated in *GmMYB181* overexpression plants. *BOP2* (BLADE-ON-PETIOLE), encoding NON-EXPRESSOR OF PR GENES1-like TFs, is important for abscission zone formation in *Arabidopsis*; *bop1 bop2* double mutation showed a series of developmental defects, including a loss of floral organ abscission (McKim et al., 2008). IDA (INFLORESCENCE DEFICIENT IN ABSCISSION), a novel putative peptide ligand; *IDA* overexpressed *Arabidopsis* plants showed early abscission of floral organs and premature cracking of siliques, indicating that the abscission zones might be responsive to *IDA* after flower opening (Stenvik et al., 2006). *MYB5, MYB61, NAP, TTG2*, and *MINI3*/*WRKY10* (Penfield et al., 2001; Garcia et al., 2005; Luo et al., 2005; Li et al., 2009; Zhou et al., 2009; Kou et al., 2012), which function in regulation of seed coat development, fruit maturation and senescence, and seed size, were down-regulated in *GmMYB181* transgenic plants. Some genes regulating *Arabidopsis* vegetative development like *WOX2, IAA31, CCD8*, and *XND1* (Schwartz et al., 2004; Sato and Yamamoto, 2008; Zhao et al., 2008; Lie et al., 2012) were significantly induced in transgenic plants. A series of genes, such as, *CRF4* (cytokinin response factor 4) (Zwack et al., 2016), *HIG1*/*MYB51* (regulates indolic glucosinolate biosynthesis) (Gigolashvili et al., 2007), *GASA5* (suppresses gibberellin response) (Rubinovich et al., 2014), *ABF3* (mediates stress-responsive ABA signaling) (Chen et al., 2013) and *ANAC055* (jasmonic acid -signaled defense responses) (Bu et al., 2008), responded to or involved in various hormone signal pathway were also significantly induced in *GmMYB181* transgenic lines. Taken together, we speculated that *GmMYB181* is likely to regulate *Arabidopsis* phenotypes via affecting the expression of corresponding genes: the DEGs related to floral organ development might cause outward curly sepals; the DEGs related to fruit/seed development possibly affected the silique size; increased lateral branches and reduced plant height of transgenic plants may be due to the expression of genes involved shoot development or hormone signaling pathway.

## Conclusion

In summary, our study elaborated the basic biological roles of *GmMYB181*. GmMYB181 is a nuclear-localized transcription activator. Overexpression of *GmMYB181* in *Arabidopsis* caused phenotype changes in floral organ morphology, plant architecture and the fruit size; and also affected the expression of 3450 genes in flower tissue. These results will provide a useful basis for our future in-depth functional analysis of *GmMYB181*.

## Author Contributions

FH and DY designed this research. HY and QX mainly conducted the experiments and analyzed the microarray data. HY wrote this manuscript. FH revised the manuscript. ZZ and JD assisted with doing the experiments. All authors reviewed the final manuscript.

## Conflict of Interest Statement

The authors declare that the research was conducted in the absence of any commercial or financial relationships that could be construed as a potential conflict of interest.

## Footnotes

1 http://www.soybase.org/soyseq/

2 http://bar.utoronto.ca/efpsoybean/cgi-bin/efpWeb.cgi

3 http://gsds.cbi.pku.edu.cn/

4 http://smart.embl-heidelberg.de/

5 http://www.ncbi.nlm.nih.gov/

6 http://www.cbs.dtu.dk/services/TargetP/

7 http://bioinfo.cau.edu.cn/agriGO/analysis.php

8 http://kobas.cbi.pku.edu.cn

9 http://bioinformatics.psb.ugent.be/webtools/plantcare/html/

10 http://planttfdb.cbi.pku.edu.cn/index.php

11 http://plantgrn.noble.org/psRNATarget/

12 http://www.soybase.org/soyseq/

13 http://bar.utoronto.ca/efpsoybean/cgi-bin/efpWeb.cgi

## Abbreviations

AD: activation domain
BD: binding domain
CDS: coding sequence
DAF: day after flowering
DAT: days after transplanting
DEG: differentially expressed genes
GO: gene ontology
HygB: Hygromycin B
KEGG: kyoto encyclopedia of genes and genomes
MS: Murashige and Skoog
NJ: neighbor-joining
ORF: open reading frame
PI: isoelectric point
qRT-PCR: quantitative real-time polymerase chain reaction
RT-PCR: reverse transcription PCR
SqPCR: semi-quantitative RT-PCR
TF: transcription factor
WT: wild type
