# Supplementary material for: GmMYB181, a Soybean R2R3-MYB Protein, Increases Branch Number in Transgenic Arabidopsis
Source: Front Plant Sci. 2018 Jul 17;9:1027. doi: 10.3389/fpls.2018.01027 (PMC6056663; doi:10.3389/fpls.2018.01027)
Supplement: Supplementary file 2 [file Data_Sheet_1.PDF]

GmMYB181, a soybean R2R3-MYB protein, increases branch number in transgenic *Arabidopsis*

Hui Yang, Qian Xue, Zhenzhen Zhang, Jingyi Du, Deyue Yu and Fang Huang*

* **Correspondence**: Fang Huang: fhuang@njau.edu.cn

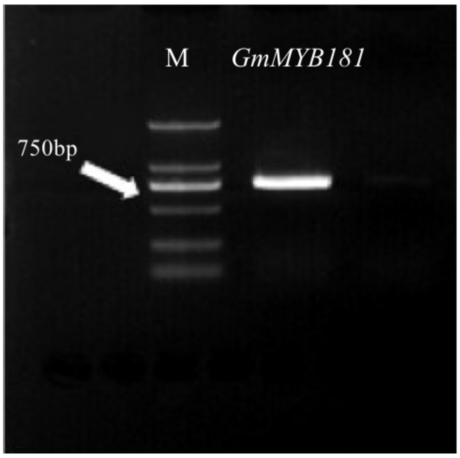

Supplementary Figure S1. *GmMYB181* cDNA PCR amplification.

M：DL 2000 Marker

```
1     atggataaaaaacaactgtgcaacacgtctcaagatcctgaagtgagaaaaggaccttggacg
       M  D  K  K  Q  L  C  N  T  S  Q  D  P  E  V  R  K  G  P  W  T
64    atggaagaagacttgatcttgatcaactatattgcaaatcatggggaaggtgtttggaattct
       M  E  E  D  L  I  L  I  N  Y  I  A  N  H  G  E  G  V  W  N  S
127   ttggccaaagctgctggtctcaaacgtaccggaaagagttgccggctaaggtggctaaactac
       L  A  K  A  A  G  L  K  R  T  G  K  S  C  R  L  R  W  L  N  Y
190   ctccgtcctgatgttagaagagggaatattacacccgaggaacaactttgatcatggagctt
       L  R  P  D  V  R  R  G  N  I  T  P  E  E  Q  L  L  I  M  E  L
253   cacgcaaagtggggaaacaggtggtccaaaattgccaagcatctacctggtaggacagataat
       H  A  K  W  G  N  R  W  S  K  I  A  K  H  L  P  G  R  T  D  N
316   gagatcaagaactattggaggaccaggatccagaagcacatcaagcaagctgagaactttcag
       E  I  K  N  Y  W  R  T  R  I  Q  K  H  I  K  Q  A  E  N  F  Q
379   caacaaattagtaataactctgagataaatgatcaccaagctagcactagccatgtttctacc
       Q  Q  I  S  N  N  S  E  I  N  D  H  Q  A  S  T  S  H  V  S  T
442   atggctgaacccatggagacctattctccaccctttatcaaggaatgttagagccattttct
       M  A  E  P  M  E  T  Y  S  P  P  F  Y  Q  G  M  L  E  P  F  S
505   tcaattcagttccccacaattaatcctgatcaatccagttgttgtaccaatgacaacaacaac
       S  I  Q  F  P  T  I  N  P  D  Q  S  S  C  C  T  N  D  N  N  N
568   agcattaactattggagcatggaggatatctggtcaatgcagttactgaacggggattaa
       S  I  N  Y  W  S  M  E  D  I  W  S  M  Q  L  L  N  G  D  *
```

Supplementary Figure S2. The coding sequence and deduced amino acid sequence of *GmMYB181*. The two MYB domains are shown in shadow, and the asterisk represents the translation termination codon.

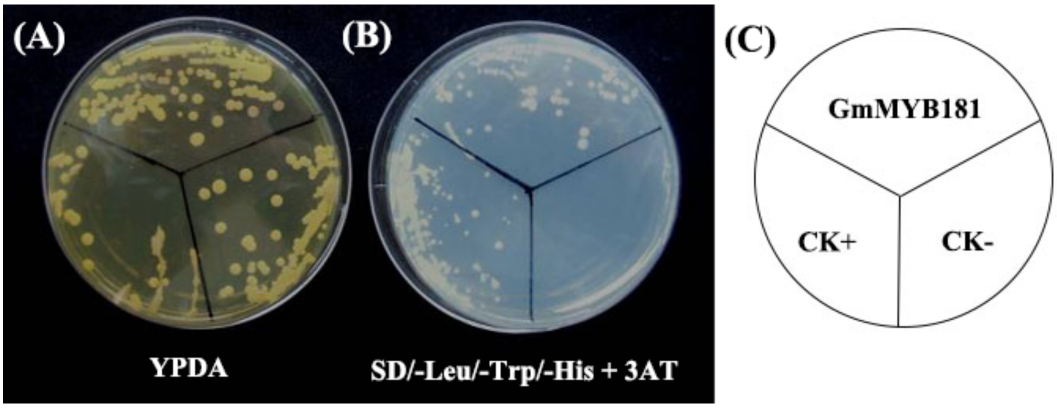

Supplementary Figure S3. Assay of GmMYB181 transcriptional activity. (A-B) Yeast cells MaV203 were transformed by BD-GmMYB181, positive (pEXP32-Krev and pExP22-Ra1GDS-wt) and negative (pEXP32-Krev1and pEXP22-Ra1GDS-m2) control, respectively. The transformants were cultured on YPAD and selective medium (SD/-Leu/-Trp/-His + 40 mM 3-AT) for examination of growth. (C) A sketch panel indicates the position of each transformed yeast strain. CK+ represents positive control, CK- represents negative control.

(A)

Download List	young_leaf	flower	one cm pod	pod shell 10DAF	pod shell 14DAF	seed 10DAF	seed 14DAF	seed 21DAF	seed 25DAF	seed 28DAF	seed 35DAF	seed 42DAF	root	nodule
Glyma16g07960	0	472	0	0	0	2	0	0	0	1	0	0	0	0

(B)

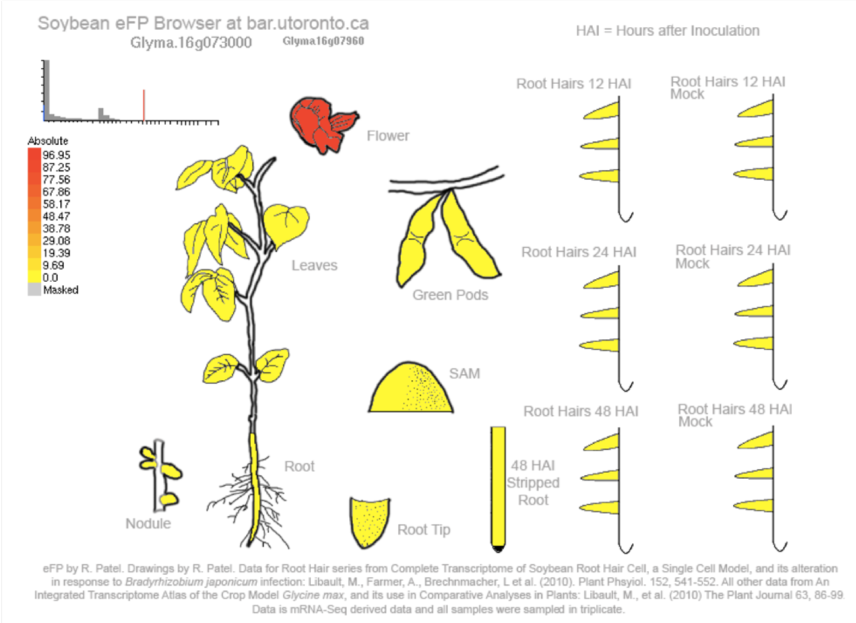

Supplementary Figure S4. Tissue expression analysis of *GmMYB181* via two databases. (A) SoyBase RNA-seq data (http://www.soybase.org/soyseq/). (B) soybean eFP Browser (http://bar.utoronto.ca/efpsoybean/cgi-bin/efpWeb.cgi).

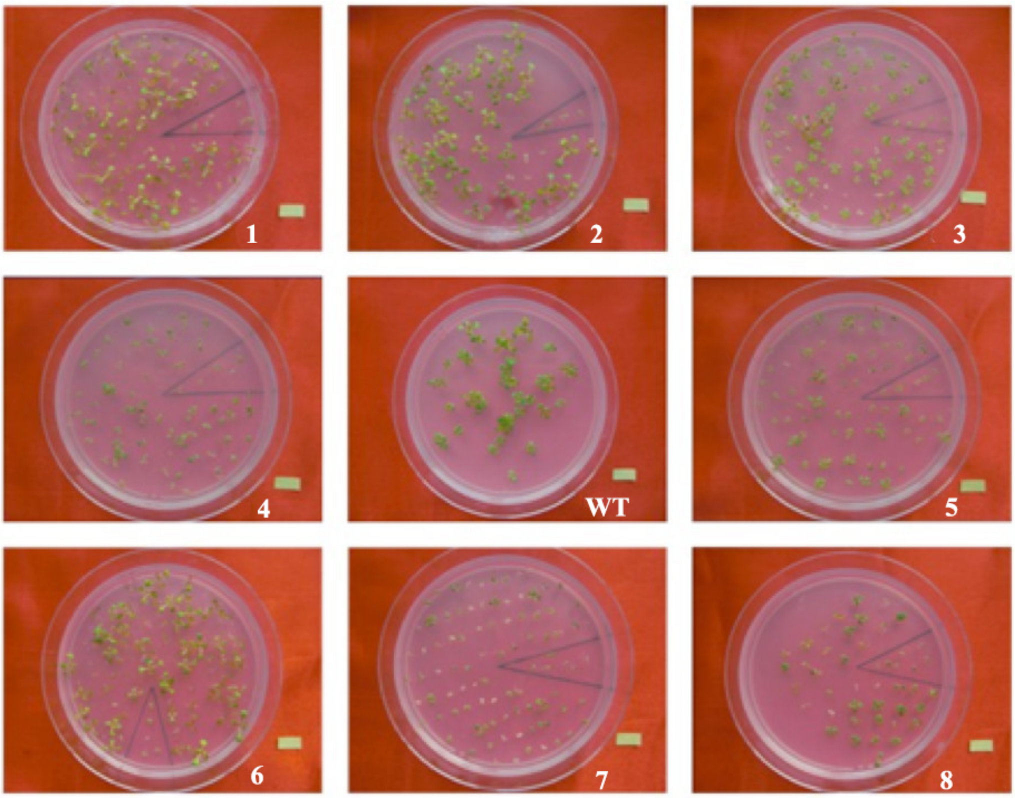

Supplementary Figure S5. Selective MS medium screening of *GmMYB181* transgenic seeds from T_1 generation. The Numerical code represents different transgenic lines. WT represents wild-type *Arabidopsis* plants (Col-0). Yellow scale bars: 1 cm.

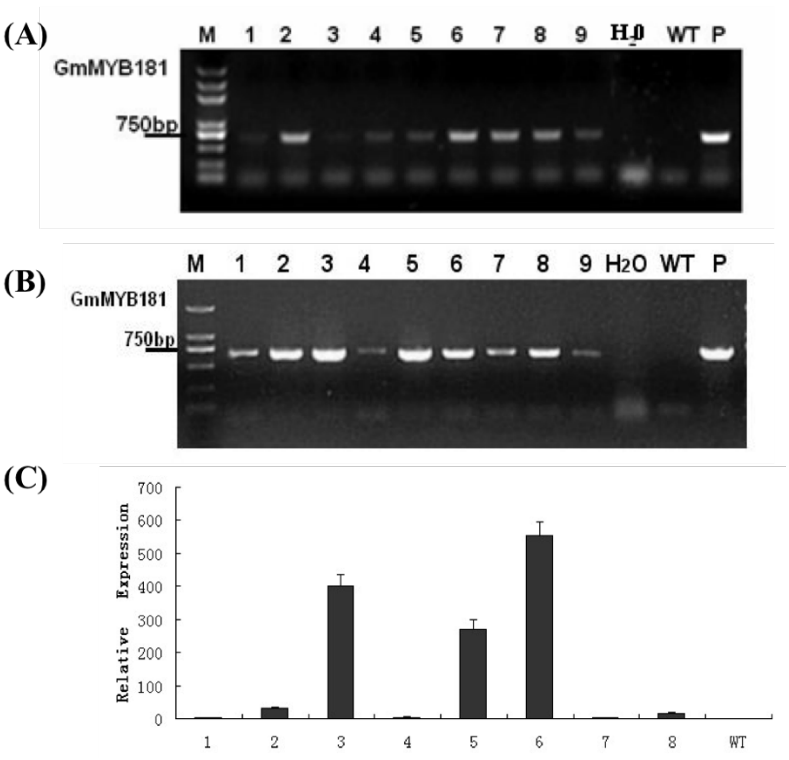

Supplementary Figure S6. Identification of *GmMYB181* transgenic *Arabidopsis* plants. (A) Identification of *GmMYB181* transgenic *Arabidopsis* plants by amplifying *GmMYB181* on genomic DNA. (B) SqPCR analysis of *GmMYB181* expression in WT and 35S:MYB181 plants. (C) qRT-PCR analysis of *GmMYB181* expression in WT and 35S:MYB181 lines. M: DNA Marker DL 5000 (in A panel) and DL 2000 (in B panel); P: positive control (pMDC83-GmMYB181 plasmid DNA); Numerical code: different transgenic plant lines; WT: wild type *Arabidopsis thaliana* (Col-0).

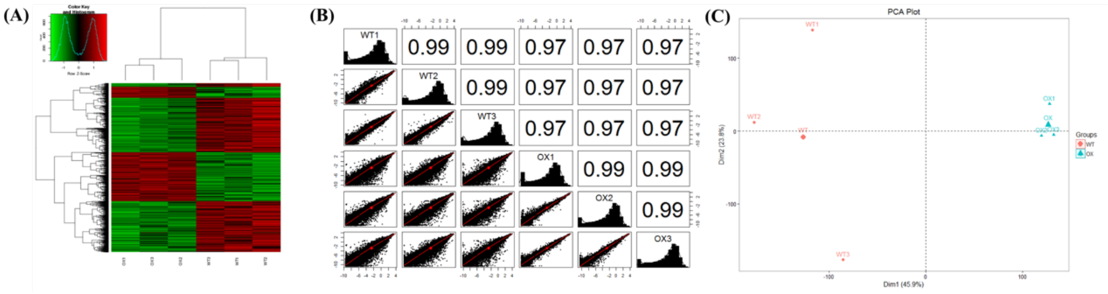

Supplementary Figure S7. Analysis of three biological replicates in microarray data. (A) Cluster heat map of differentially expressed probe sets by using Cluster 3.0 and Java TreeView 1.1.6. (B) Correlation analysis of gene expression in different samples. The closer the Pearson coefficient is to 1, the higher the correlation of the two samples. The picture was drawn by local R script. (C) PCA analysis of gene expression in different samples. Dim1 and Dim 2 are the two principal coordinate components, Dim1 represents the principal component of the data that maximizes the variance of the data, and the percentage indicates the contribution of the principal component to the difference. Each point in the graph represents a sample, and different colors represent different samples. The picture was drawn by local R script.

Supplementary Table S1 Primer pairs used in soybean.

Gene Names	Sense primers (5'-3')	Antisense primers (5'-3')	Function
GmMYB181	AGTATCTCTCTCTCTCCCCT	AAGCCAGAGACATTCCAGTG	cDNA isolation
GmMYB181	ATGGATAAAAAACAACTGTGCAA	TTAATCCCCGTTCAGTAACTGC	CDS isolation
GmMYB181	GGGGACAAGTTTGTACAAAAAAGCAGGCTATGGATAAAAAACAACTG	GGGGACCACTTTGTACAAGAAAGCTGGGTATCCCCGTTCAGTAACTG	Transformation
GmMYB181	GGGGACAAGTTTGTACAAAAAAGCAGGCTATGGATAAAAAACAACTG	GGGGACCACTTTGTACAAGAAAGCTGGGTATCCCCGTTCAGTAACTG	Subcellular localization
GmMYB181	GGGGACAAGTTTGTACAAAAAAGCAGGCTATGGATAAAAAACAACTGTGCA	GGGGACCACTTTGTACAAGAAAGCTGGGTTTAATCCCCGTTCAGTAACTG	Transactivation activity
GmMYB181	ACACTGGAATGTCTCTGGCTTAAA	TTACAATGCACATAGCATCTCATTTG	SqPCR
GmMYB181	ACACTGGAATGTCTCTGGCTTAAA	TTACAATGCACATAGCATCTCATTTG	qRT-PCR
GmActin	TTTGCTGGTGATGATGCT	ACCTCTTTTTGACTGGGC	SqPCR
GmTubulin	GGAGTTCACAGAGGCAGA	CACTTACGCATCACATAGCA	qRT-PCR

Supplementary Table S2 Primer pairs used in *Arabidopsis*.

Gene Names	Sense primers (5'-3')	Antisense primers (5'-3')	Function
AtTubulin	CTCAAGAGGTTCTCAGCAGTA	TCACCTTCTTCATCCGCAGTT	qRT-PCR/SqPCR
GmMYB181	GGGGACAAGTTTGTACAAAAAAGCAGGCTATGGATAAAAAACAACTG	GGGGACCACTTTGTACAAGAAAGCTGGGTATCCCCGTTCAGTAACTG	PCR analysis/Sq-PCR
GmMYB181	CCTGGTAGGACAGATAATGAGATCAA	AGTTATTGCTGATTTGTTGCTGAAA	qRT-PCR
BAM2/A_84_P12759	GGAACTCTGCCGCCGAATA	CGACCCGTTAAGGAAATTCTCA	qRT-PCR
SOBIR1/A_84_P18180	CGTTCTTCGGCCAGTGATGT	TCGTGACACGGAGAACGTATTC	qRT-PCR
BOP2/A_84_P291394	GACGTGACTTTCAGCGTTGAAG	AGTCTGTCCCACAAAAGAATTTGC	qRT-PCR
IDA/A_84_P610349	TCGCGGCGAGTAGTTCTTGT	GGAATGGGAACGCCTTTAGG	qRT-PCR
IDL1/A_84_P612395	TGTTTTCCTCTTGATTTTTGGTTCA	AAAACCCCCAATGATCTCTTGTC	qRT-PCR
IDL3/A_84_P768403	TTGTCCTTTTGAGTTGCTGCAA	TGATCATGAGGTGGCGAAAC	qRT-PCR
PGAZAT/A_84_P861846	TCATGCAAACGGAACAAAGCTA	AATCTGGATCTGCTGTGCATTTC	qRT-PCR
PUCHI/A_84_P20594	CGTGTGATCTCTCTGCCATGA	GCAACGACATCTCCAAAAGCA	qRT-PCR

Supplementary Table S3 plant *MYB* genes' information.

Gene name	Accession number
MtR2R3-MYB	XP_013450781
GtMYB2b	BAF96932
VvMYB24	ABW34394
AtMYB21	AEE77366
AtMYB24	AED94536
AtMYB118	AEE77363
TaMYB26	JF951909
SbMYB42	AAL84762
LgMYB21	BAL41446
